# Editorial Correspondence

**Published:** 1857-03

**Authors:** W. F. Westmoreland

**Affiliations:** Paris


### EDITORIAL AND MISCELLANEOUS.

### EDITORIAL CORRESPONDENCE.

Paris, December 7th, 1856.

*Dear Doctor*—Exclusive systems of practice in medicine, and methods of operating in surgery, or perhaps I should be better understood were I to say, hobbys in the practice of medicine and surgery have done as much to retard the progress of science as any one consideration. All combat the principle, yet all are to some extent culpable, and worse; it is usually those who have position and influence that are most persevering in forming the ories, and proposing operations, and for a series of years, bending facts, however stubborn, to suit and support their peculiar views. No one who is the least familiar with the history of medicine and surgery will, for a moment, doubt the truth of this proposition. Let us then all be upon the alert, watching ourselves as well as our neighbor, and if we should see any thing rational attended with happy results, to whatever extent we may be enraptured with our own peculiar views, let us not pass it by as unworthy of consideration, but stow it away until circum stances shall make it practicable. There are accidents and deformities requiring operations which, in different cases, differ so much in extent, position, &c., that no one method of operating, however successful in the majority of cases, can be applied with the same happy result in all. Vesico-vaginal fistula, which is at present exciting considerable attention, and for which several new methods of operating have been proposed within a few years, is an example of this class of accidents. In this loathsome lesion, as in many others, it is evident that no one method can be alike applicable in all cases—that the operation proposed should be dictated by the position and extent of the lesion, and other considerations surrounding the case, rather than by our predilections for any favorite method; and that the greater the number of successful methods proposed, the greater will be our resources. With this view, I have thought it not amiss to record here the method adopted in a case, some time since, by M. Nelaton, which although not new, (the figure of eight suture,) still the manner of introducing and extracting the pins is certainly worthy of consideration.

The fistula was small, rather transverse, extending more to the right than to the left of the median line, nearer the neck of the bladder than the neck of the uterus. After bringing down the neck of the uterus with Museux's forceps, the os externum being previously dilated, the fistula could be readily seen; the edges were now freshened with considerable loss of substance, removing the entire cicatrix—a procedure which M. Nelaton considers essential to the success of the operation. Three ordinary suture pins, with a thread firmly attached to the head of each, were now introduced as in an ordinary wound, and the freshened edges brought accurately in contact by means of a ligature passed around the pins in the form of a figure 8. The pins were so directed that the head of each pointed towards the mouth of the vagina, so that by means of the threads attached which passed out of the vagina, they could at any time be extracted without distending the vagina, or otherwise interfering with the wound. A catheter was secured in the urethra. On the fifth day after the operation the first pin was removed by means of the thread attached, two days after the two remaining pins were removed in the same way; the urine all the time passing through the catheter. Two weeks after the operation, the catheter was removed, when it was found that she could, for a length of time, retain her urine, passing the whole per urethra—demonstrating the entire success of the operation. Upon examination sometime after, it was found that the wound had united regularly in its whole extent. It is evident that this method cannot be adopted in all cases, but when applicable, is certainly rational, and from the above result, may be performed with success.

Since my last letter, we have had, in Paris, the "Blue man," Butler, of New York; he, for several days, occupied a bed in the wards of M. Nelaton, and during his stay was the centre of attraction. Butler, as you are apprised, is a striking example of the discoloration of the skin by the long and continued use of nitrate of silver, administered in his case with the hope of relieving an inveterate epilepsy, with, however, no very favorable result, as he is at present in Europe to be treated, not for the discoloration of the skin, with which I believe he is rather pleased, but for his epilepsy. This case certainly proves the permanency of such discolorations, as it has for eighteen years resisted all attempts to remove the deposit. Butler, it appears, has been submitted to quite a variety of plans of treatment for the relief of his epileptic attacks; more recently, Dr. Green, of New York, cauterized the larynx and trachea by means of the probang, with no favorable result. Dr. Parker, of the same city, proposed castration, the proposition, as Butler says, having for a basis the favorable result of some cases operated on by a physician of Georgia, whose name I can not at present recall—you, however, I am confident, will recollect the report. Butler crossed the Atlantic to place himself under the care of Marshall Hall, who saw him during his recent visit to America, and proposes to cure his epilepsy by tracheotomy. Upon his arrival in London, and finding Dr. Hall absent for some length of time, he visited Paris to have the opinions of some of the most prominent physicians. Not finding M. Nelaton disposed to favor any operation, but to experiment upon the deposit, he left the hospital and Paris, for London, I suppose.

The discussion at the *Académie de Médicine*, upon the treatment of ovarian dropsy, so frequently refered to in my letters, has been closed, not before, however, all the members of that savant body, who were disposed, had expressed their views upon this complex proposition. As remarked in a previous letter, it is not convenient here for me to follow this discussion in all its meanderings, and were I to attempt it, I doubt very much whether I should render the subject less complex—whether I should not, by recording the diversity of opinions, add to the confusion which already exists. Much has certainly been said, both for and against the interference of the surgeon, and much upon either side to no purpose. As in almost all discussions, there has been two extremes, both, perhaps, alike irrational—the more practical occupying a happy medium. It is evident, however, that the old doctrine of non-intervention, if you will allow the expression, as regards the radical treatment, has received a blow from which it is not likely ever to recover.

M. Velpeau closed the discussion; his remarks, upon that occasion, were entirely practical, and was certainly the best solution of the whole subject that has been given during the discussion; so important do I consider this last effort, that I give it below (in substance) at the risk of worrying you with this subject. That it may be better appreciated, I propose before doing so, to make an extract from a memoir read before the *Académie* by M. Creuveilhiér. In this memoir we find the following in regard to the varieties of ovarian tumors:

" Cysts of the ovary do not always constitute the same anatomical lesion. The question of treatment which should always be surgical, will depend to a very great extent upon the character of the cyst, as regards the liquid it contains, the disposition of its walls, and its structure. As to the liquid they contain, they may be divided into *serous* cysts, containing limpid serum or sérum of various colors; into *albuminous* cysts, in which the liquid resembles the white of an egg; and into *gelatinous* cysts, the contents resembling jelly; these distinctions, although extremely important in a therapeutical point of view, are not always easily established by the character of the fluctuation.

As regards the disposition of the cysts themselves, they may be divided into four varieties: firstly, *unilocular* cysts; secondly, *multilocular* cysts; thirdly, *areolar* or *vesicular* cysts; fourthly, *compound* or *complicated* cysts. The latter being the result of the association of a unilocular with a multilocular cyst, or either of these with the areolar or vesicular; again, a cyst may be considered compound or complicated, when it has for its basis, a fibrous tumor of the ovary."

The above is perhaps the most complete as well as most practical division of ovarian cysts. It is true that several of them are extremely rare, yet all have been recognized.

M. Velpeau's conclusion was in substance as follows:

"The question at present under discussion before the *Académie* is extremely complex. For its solution, it is necessary to determine, firstly, the ordinary duration of the disease; secondly, its gravity; thirdly, the value of the various plans of treatment to combat it: that is, to determine, for example, whether a radical cure ever follows the simple puncture, and in what proportion of cases, and if the treatment by injections be proposed, to determine whether iodine is better than all others; and fourthly, to be able to distinguish the various cysts.

"The first proposition,—the duration of ovarian cysts—can it be determined? What has been said here in regard to their duration is far from being exact. Two, six and ten years have been given as their term of duration; how have they arrived at such conclusions? At the commencement,—that is to say, when the cyst has but the size of an egg or an orange,—it is seldom recognized; and often, when recognized, and the physician called, it is extremely difficult to determine the date of its origin; again, are not all apprised of the very great difference in the march of serous collections? In dropsy of the tunica vaginalis, for example, there are some cases that acquire a development in six months that others are six years in attaining. In some women, again, ovarian tumors are recognized much earlier than in others; thus, it is evident that in a delicate woman, with thin abdominal walls, a cyst would be much easier and earlier detected than in a corpulent woman, with thick abdominal walls. It is, then, extremely difficult to determine the ordinary duration of such cysts. I have seen ovarian tumors the size of a large orange, acquire, in less than a year, the dimensions of the head of an adult, and after arriving at that point, I have known quite a number of women to live four, ten, fifteen and eighteen years. Taking all into consideration, I am of the opinion, that, in the majority of cases, women attacked with this form of dropsy, may live six, and perhaps even more than eight years after the tumor is of sufficient size to be detected; and since, in a number of cases, life is prolonged, without the intervention of any treatment whatever, to fifteen and eighteen years, it would be extremely imprudent to adopt any plan of treatment that would be attended with great danger. But, as it must, sooner or later, prove fatal, it is certainly rational to interfere."

Arriving at the third proposition, M. Velpeau adds: "Can cysts of the ovary be cured by the administration of internal remedies? The negative response of MM. Cruveilheir and Trousseau greatly surprised me, as I am certain that I have seen cases cured in this way. The idea has been advanced that ovarian cysts, from their isolation from the organism, were not under the influence of internal remedies; it has been asked, how it was possible, under such circumstances, for absorption to take place. I might ask, also, if the organism, under such circumstances, secreted such large quantities of liquid, why it is, that, under the same circumstances, it might not be absorbed. This is a question, however, that cannot be determined except by facts. Hydrocele, although very rarely, it is true, sometimes presents examples of spontaneous cures.

M. Velpeau here mentioned a case of hydrocele, in which he proposed an operation, which disappeared spontaneously in less than forty-eight hours.

When the cysts, adds M. Velpeau, are very small, this spontaneous or accidental rupture may result in a radical cure, as has been cited in several cases during the discussion ; but often death, is the consequence. In a report of 72 cases of such ruptures, by Tilt, there were thirty deaths ; making it, by no means, a desirable accident—it is not, however, very frequent. I have only observed it twice, and in both cases death was the result. At the commencement of this discussion, I mentioned some cases in which the simple puncture was followed by death. I had no idea at the time these facts were mentioned, that they would occasion the long and deplorable statistics that have been produced in this discussion.

According to Southom, as cited by M. Trousseau, in twenty-one cases of simple puncture, there were four deaths during the first twenty-four hours, three during the first month, and fourteen during the first year. In thirty-six cases reported by Lee, there were three deaths in the first twenty-four hours, six during the first few days, twelve in one year, five in three years, one at the end of six years, and one at the expiration of fifteen years. In a report by Kiwisch of sixty-four cases of simple puncture there were nine deaths during the first twenty-four hours, six after a second puncture, fifteen after the third, fourth, fifth or sixth puncture. It would appear from the above, that the simple puncture is extremely fatal in England and Germany; something which I must say, in all candor, does not appear to me possible. We have never seen any such results in France. I said that I had seen four deaths from the simple puncture; they were cases very grave and complicated—gelatinous and multilocular cysts—but no one of them died in twenty-four hours; all lived for some length of time. Within the past thirty years, I have performed this operation quite a number of times, and with the exception of the four cases above-mentioned, have never seen death as the result of the puncture—all have lived four, five, six, and ten years, some few as many as fifteen years. This, as you see, resembles, in no particular, the frightful statistics above alluded to. Why is this? Statistics are frequently on the order of Æsop's Fables; in which is found all that may be desirable for good or bad. It is more than probable, that the statistics above referred to, are not exact; I shall not, however, attempt here their solution. After a review of all that has been said, I am still firmly of the opinion, that the simple puncture within itself, is not fatal, and is generally inoffensive. Will it cure ovarian dropsy? Some cases; I saw one example, several years ago, in connection with Récamier and Nelaton. The simple puncture like the spontaneous rupture, sometimes results in a cure, but it is necessary to say, that such cases are exceptional. Has it no inconveniences? It certainly has. Where we are forced to resort to it frequently, for the relief of urgent symptoms, we, in the end, exhaust the patient. It should be resorted to rarely, and with decision.

The extirpation of the cyst! There is a singular contradiction between the idea, that is, with reason advanced, of the extreme gravity of the operation, and the statistics of the English and American surgeons. I shall not attempt here to explain this contrast. It is very certain, that I shall never dare perform such an operation; and I should here say, in honor to the surgeons of France, that this operation has never found favor with us.

I come now to the treatment by the injections of iodine. This method is not as new as is generally believed—the injection of cysts was practiced during the past century. How is it, then, that this method is creating at present, such an emotion? It is natural we should apply to cysts of the ovary, what has been applied to hydrocele; this is exactly what has been done. As the liquid, that was first employed in the treatment of hydrocele, was extremely irritating, it frequently happened that the inflammation went beyond the limits that were then thought necessary to insure a cure, and resulted in extremely grave accidents. This prevented the generalization of such injections. But, as it was demonstrated to my satisfaction, that for the cure of such collections, it was not necessary to determine in the walls of the cyst, a high grade of inflammation, and, that iodine, which is much less irritating than wine, produced sufficient irritation to obtain the result desired, without the risk of accidents so frequent from the injections of alcoholic liquids, I saw at once, the advantages that would result from applying the treatment of hydrocele to the various cysts, and thus multiplying its use. This question, besides, is not new; it was discussed here in 1846—I then expressed my views before the *Académie* upon this point; they were supported by M. M. Bérard and Jobert, and combatted by Blandin, Gerdy and Roux. It was at this epoch, that several surgeons commenced studying this subject, and that M. Boinet commenced his experiments, which he has multiplied and studied with such zeal and care, that this method has been recognised as his—bearing his name.

In the practice of M. M. Boinet, Robert, Monod, Demarquay, Huguier, Briquet, Nelaton and in my own, we have a total of 130 cases of ovarian dropsy, treated by the injections of iodine. Let us see what we will have from the analysis of 130 operations. In this number, there were thirty deaths and sixty-four cures. Thirty deaths in 130 operations! This is certainly a very heavy per cent., and I would be little disposed to defend the operation, did I believe that such a per cent. of deaths would necessarily result. Let us go still further and determine the circumstances, if possible, that produced this result. By what accidents did these thirty women die? When I performed the first few operations of this kind, that which occupied me all the time, and sometimes arrested me, was the danger of injecting such a vast cavity, with the great difficulty in determining the character of the cyst upon which I was operating. It has been said here, that the diagnosis of ovarian tumors is attended with no difficulty. My compliments to any of my confrères who are of this opinion. As to myself, I must say, that I have found the diagnosis, in a number of cases, extremely difficult. When these cysts are small, it is certainly very difficult, if not impossible, to distinguish the true cysts of the ovary, from other cysts or collections of liquid in that region, as cysts of Wolfeian bodies, extra-uterine pregnancy, circumscribed dropsy of the peritoneum, &c., &c. When the cyst is sufficiently large to overcome this confusion, and it being recognised without difficulty, there is something still of great importance to determine—to consider the character of the liquid it contains. All know the difference in point of gravity, and curability between a serous and a gelatinous cyst. Not only does the liquid differ in different cysts, but I have demonstrated that in the same cyst—we may have at different examinations a liquid which differs greatly in aspect. Thus, a serous cyst to-day, may later become sanguineous, and vice versa. Besides, I have seen cysts in which the first liquid extracted presented the color of blood, and later, the cyst becoming distended, the liquid was serous. It was the result, most probably, of the injection of iodine—thus transforming its walls. I have often had an opportunity of witnessing this transformation in hydroceles—to see a hydrocele, in the liquid presented; the color of blood, to become later serous. I have, by observing such cases, learned to produce this transformation artificially. I have often cured hydroceles of the above character, by converting them, by means of the first injection, into an ordinary hydrocele.

The operation by the injection of iodine, is it attended with danger? What are the dangers? I must acknowledge, that I, at one time, dreaded greatly the inflammation of the cyst; but I have since learned that it is not to be feared. The inflammation is never intense; and is always limited to the point the liquid touches. Neither do I fear the puncture, as I have learned from experience, that the simple puncture is not dangerous.

Where is the danger then, and how can we explain the development of fatal accidents? My impression is, that the accidents are the result of the employment of the canula or tube, as practiced by some surgeons. The method of operating in which the canula or tube is left in the cyst, is certainly objectionable, as it is almost always attended with a suppurative inflammation of the walls of the cyst. In the statistics of this operation, the methods of operating have been confounded; there is, however, a very great difference; for example, between the method by canula or tube, and the subcutaneous process; a difference which satisfactorily explains the difference in results. In the subcutaneous method of M. Guerin, there is rarely ever suppurative inflammation, while in the process with canula, there is, as above suggested, almost always suppurative inflammation, which, in the majority of cases, is kept up until the patient is exhausted, notwithstanding the repeated injections of iodine.

Let us see, now, what were the methods adopted in the thirty cases before mentioned, that proved fatal. In twenty of the cases a canula was left in the cyst, if I am not mistaken; then, we may very well attribute the unfavorable results in the twenty cases, to this circumstance. There are only ten cases then, where death may be attributed to the injections of iodine. Ten deaths in 130 operations is not such a great per cent.; it is certainly a very satisfactory result in any new operation, where there is such hesitation in its performance. When, then, it is demonstrated that the treatment of ovarian cysts, by the injection of iodine, is not more fatal than the simple puncture, with many more chances to cure, it must be accepted as a method with many advantages.

In resumé, then, I will conclude this subject by saying, that cysts of the ovary, the most frequently mortal in a length of time, extremely variable, but which may be approximated at a few years, say six or eight, one but little affected by internal remedies, may rupture spontaneously; but such ruptures, although sometimes followed by a cure, is ordinarily very fatal. That the simple puncture does not offer, within itself, any great danger, but that it is to some extent, objectionable, where it is for a long time, and at short intervals repeated—as under such circumstances it exhausts the patient—that the proposition to extirpate such cysts should be rejected—that the methods by injection, although dangerous where irritating liquids were used, have become inoffensive, and of great advantage since iodine has been adopted—that the injections of iodine is, without doubt, of great utility in *serous* cysts. As to the other forms of cysts, the wisest plan is to let them alone.

PARIS, DECEMBER 20th, 1856.

In a letter a few weeks ago, speaking of the treatment of syphilis by means of numerous inoculations with the pus from a primitive chancre, or in other words, by *syphilisation*, I promised to keep you informed of the progress of a case in the wards of M. Nelaton, which is being submitted to this new treatment. The inoculations have been constantly going on—the patient at present presenting between eighty and one hundred chancres. The idea of such a number upon one patient would, without further explanation, appear frightful, and would at once lead us to conclude that, aside from the disgust and suffering, which we would imagine great, under such circumstances, there would be more or less constitutional disturbance, neither of which is the case—the patient appearing more comfortable, and certainly in better spirits than before the commencement of the experiments. From the first crop of chancres, which were large, the successive crops have gradually diminished in size, until at present they are not larger than a grain of wheat, it being with difficulty that the inoculations take effect. As to the actual condition of the patient, there has been no very marked change, the most important being, the disappearance of osteocope, which, before the commencement of the inoculations, existed to such an extent as, frequently, to prevent the possibility of sleep. The nodes on several of the superficial bones have, perhaps, to some extent, diminished in size. It is evident that the disease is making no progress, and, from all the symptoms, would appear to be yielding to its treatment. It is impossible, however, to speak positively upon this point, as in a few weeks we may see the disease recommence its ravages. As soon as the inoculations, which are at present made with the pus from previous inoculations, entirely cease to take effect, they will be recommenced, as at first, with pus from a primitive chancre. This case is watched with great anxiety by the friends of the doctrine of syphilisation, as a favorable result would place them entirely beyond the reach of their adversaries, at the head of whom is M. Ricord.

I visited the wards of M. Maisonneuve a few days ago, to witness an amputation of the leg, without the use of saw or knife—a novel proposition, you will readily agree. I, however, only saw the operation upon the dead subject, and after witnessing it, was thoroughly convinced that such operations should be confined strictly to the dead-room. The operation consists in first breaking the bone by means of a machine which I shall not attempt to describe, and afterwards to make a section of the soft parts, a few inches below the fracture, with the *ecraseur*, so much vaunted by M. Chassaignac for the extirpation of polypus of the uterus, hemorrhoids, &c., consisting in a chain or piece of wire passed through a metalic box and tightened by means of a screw, thus dividing the tissues by constriction; after the complete division of the soft parts, the limb is detached by forcibly tearing the bone from the muscles. One of the objects in this plan of amputating, is to prevent the loss of blood, he contending that there is no hemorrhage from the division of the tissues, however vascular, and even of large sized arteries, with the *ecraseur*. There are other considerations which he did not discuss, and which I shall defer until I see the operation upon the living subject, which will likely not be a great while. He has amputated an arm by this process —the patient died, however, in a few days after the operation.

There are, at present, in the wards of M. Velpeau, four cases of fracture, which, from their rarity and other considerations, are not without interest. The first case that I shall notice, and which is the least frequent of all, is a fracture of the internal portion of the glenoid cavity,—the result of a fall, which, at the same time, produced a dislocation of the humerus downwards and forwards, the fracture being produced by the force with which the head of the bone was driven against the internal lip of this cavity. There is always a dislocation of the humerus accompanying this fracture, the most prominent symptom being a reproduction of the dislocation from the slightest motion; crepitation is possible after the reduction of the dislocation. The treatment consists in fixing the head of the humerus in its proper position for fifteen or twenty days. M. Velpeau contends that this fracture is not so rare as is generally supposed; that its existence is frequently not recognized by the surgeon.

Another case in the same locality, and also very rare, is a fracture of the anatomical neck of the humerus—an intracapsular fracture of the neck, the solution of continuity being entirely above the insertion of the capsule—the head of the bone resting without attachments in the capsule. If there be no great muscular developments, nor impaction, the fracture is readily recognized by the mobility of the detached head, crepitation, &c. What should be our prognosis in such a fracture? Will we have union of the two fragments, or necrosis of the detached head? M. Velpeau contends that if the humerus be kept fixed in a favorable position, that in quite a majority of cases, the functions of the limb, to a great extent, at least, will be restored in a few weeks. The case under consideration is in support of M. Velpeau, as three weeks have past without any accident; the patient appearing to be doing as well as could possibly be expected. Notwithstanding the views of M. Velpeau, and the favorable result of the above case, I am disposed to believe, as Prof. Erichsen has suggested, that when we have such favorable results, that there is either an impaction of the fragments, or else the fracture at some point, at least, has descended below the insertion of the capsule, the superior fragment receiving its support from this membrane.

The other two cases, a transverse fracture of the patella, and a fracture of the olecranon process, are comparatively much more frequent, but from the various plans suggested to retain the fragments in contact, are not less interesting. The fracture of the patella, as above suggested, is transverse, the result of a fall, the knee coming in contact with the curb-stone. After discussing the modes of union of this fracture, and the value of the various kinds of apparatus proposed for its treatment, M. Velpeau concluded by saying, that although there are upon record several well attested cases of osseous union, that as a general rule the union is ligamentous, and that we are not authorised to attempt, by extreme measures, to produce what experience has proved exceptional—contending that there is but little, if any difference, as regards the functions of the limb, whether the ligament uniting the two fragments be a quarter or half an inch long. He says that the most important consideration in the treatment of this fracture is position —the leg extended, and the thigh flexed upon the trunk. In the case under consideration, the limb was placed in a wire gutter, and the superior fragment brought down by means of a bandage attached.

The case of fracture of the olecranon process, is being treated by a straight splint and the figure of eight bandage.

Since writing the above, I have had an opportunity of examining an extremely rare lesion, which, as I am in the way of speaking of rare accidents, I will record here: It was a dislocation forwards, of the superior extremity of the middle piece of the sternum, in a man between forty and fifty years of age. It has been contended by some surgeons, that this accident is not observed except in young subjects, and, as the result of a direct force; this case, however, is in direct opposition to such an opinion, as the subject has already passed the middle age; and from the history of the accident (the patient falling from the second story of a house, lighting on his feet, and breaking, at the same time, both legs), as well as the complete absence of contusion of the soft parts covering the sternum, there is not the least doubt but that it is the result of an indirect force. But, how shall we account for a dislocation of the sternum, by an indirect force? Shall we look upon the sternum in such cases, as a sort of second vertebral column, receiving a portion of the shock? Or, shall we account for the dislocation under such circumstances, by the chin coming in contact with the superior portion of the sternum, and thus transmitting the shock, as some have imagined? We can conceive of the possibility of the chin transmitting the shock, if the subject light upon the head, but must say, that it requires a stretch of the imagination to account for the accident in this way, where the subject lights upon his feet.

Yours, &c.,

W. F. WESTMORELAND.

